# Systematic Analysis of Plasma‐Derived Extracellular Vesicle‐Enriched Samples in Age‐Related Macular Degeneration Reveals Oxidative Stress Linked to Altered Lipid and Protein Profiles

**DOI:** 10.1002/jex2.70178

**Published:** 2026-08-29

**Authors:** Marzena Kurzawa‐Akanbi, Jennifer Haggarty, Carina Hansohn, Mohamed T. Patel, Angela J. Cree, Peisi Teo, Milly Armstrong, Nathan Coles, Mark Platt, Helen Griffiths, Heather J. Cordell, Ahmad Khundakar, Pola Goldberg Oppenheimer, Phillip Whitfield, Andrew Lotery, Henning Urlaub, Sina Mozaffari‐Jovin, Majlinda Lako

**Affiliations:** ^1^ Biosciences Institute, Faculty of Medical Sciences Newcastle University Newcastle Upon Tyne UK; ^2^ NIHR Biomedical Research Centre: Newcastle Newcastle Upon Tyne UK; ^3^ MVLS Shared Research Facilities, College of Medical, Veterinary and Life Sciences University of Glasgow Glasgow UK; ^4^ Max‐Planck‐Institute for Multidisciplinary Sciences Göttingen Germany; ^5^ School of Chemical Engineering, College of Engineering and Physical Sciences University of Birmingham Birmingham UK; ^6^ Clinical and Experimental Sciences, Faculty of Medicine University of Southampton Southampton UK; ^7^ School of Health and Life Science Teesside University Middlesbrough UK; ^8^ Loughborough University Loughborough UK; ^9^ Population Health Sciences Institute Newcastle University Newcastle Upon Tyne UK; ^10^ Institute of Clinical Chemistry University Medical Centre Göttingen Göttingen Germany

**Keywords:** age‐related macular degeneration, biomarkers, extracellular particles, extracellular vesicle‐enriched samples, lipidomics, plasma, proteomics, Raman spectroscopy

## Abstract

Vision impairment caused by age‐related macular degeneration (AMD) is a global health priority. Retinal pathology is driven by ageing and exacerbated by genetic and environmental risk factors. Peripheral blood circulation is increasingly recognised as a contributor to disease initiation and progression, and a source of biomarkers to support earlier diagnosis and personalised treatment strategies. Here, we performed an analysis of plasma‐derived extracellular vesicle (EV)‐enriched samples from 30 AMD and 30 controls. Proteomics identified significantly altered proteins in AMD, the majority of which were downregulated with a strong interaction network involving complement proteins and endopeptidase inhibitors. Importantly, many of the altered proteins are known AMD biomarker candidates and act in pathways affecting AMD: oxidative stress response, immune function and proteolysis dysregulation. Lipidomics revealed an increase in total sphingomyelin to ceramide ratio in AMD, indicating lipid metabolism defects. Raman spectroscopy complemented these findings by demonstrating protein and lipid oxidative modifications, alongside compositional abnormalities in AMD samples. Thus, AMD plasma EV‐enriched samples carry a systemic signature of complement and coagulation dysregulation, impaired redox homeostasis, and altered sphingolipid metabolism, reflecting established mechanisms of AMD retinal pathology. These AMD‐associated biochemical profiles form a promising source for developing new diagnostics and mechanistic insights for precision medicine.

## 1 Introduction

Irreversible vision impairment due to age‐related macular degeneration (AMD) is a global public health concern. The number of individuals with vision impairement due to AMD more than doubled between 1990 and 2021, reaching an estimated 8.06 million, and is projected to reach 21.34 million cases globally by 2050 (Collaborators 2025). This notable increase in prevalence can likely be attributed to population ageing and growth. AMD is widely recognised as a multifactorial disease; increasing age, polymorphisms in and near complement genes (Fritsche et al. 2016) and an MHC class II risk haplotype (Gorman et al. 2024), alongside environmental risk factors such as smoking (Khan et al. 2006) and diet (Angelia et al. 2024; Thee et al. 2023), are associated with a higher risk of developing the condition. Notably, health equity and access are key contributors to the rising overall burden of AMD, highlighting the need for public health initiatives to reduce the effects of modifiable risk factors, such as smoking, alongside the implementation of early cost‐effective disease detection and treatment options (Collaborators 2025).

Clinically, AMD is defined based upon the presence of lesions within two‐disc diameters of the fovea (the central part of the macula) in either eye visible on colour fundus photography. These lesions include medium (>63 µm and ≤125 µm) or large (>125 µm) drusen deposits, retinal pigment epithelium (RPE) pigmentary abnormalities or the presence of changes associated with late‐stage AMD: neovascularisation or geographic atrophy (Ferris et al. 2013). Multimodal imaging, including optical coherence tomography, has facilitated the recognition of subretinal drusenoid deposits (also known as reticular pseudodrusen) as a distinct entity from conventional drusen, representing aggregations of material in the subretinal space between the RPE and photoreceptors (Spaide et al. 2018). Pseudodrusen are associated with an increased risk of developing late AMD lesions. Similar to typical sub‐RPE drusen (Laine et al. 2007; Crabb et al. 2002; Wang et al. 2010), pseudodrusen deposits contain protein components, such as apolipoprotein E, complement factor H (CFH) and vitronectin (Rudolf et al. 2008). There seems to be, however, a difference in their lipid composition; drusen contain both unesterified and esterified cholesterol (Curcio et al. 2011), while only unesterified cholesterol has been detected in pseudodrusen (Rudolf et al. 2008). Additional differences in mineral content between the two deposit types indicate distinct biogenesis pathways rather than mislocalisation of RPE‐derived debris (Spaide et al. 2018). Accordingly, Spaide et al. proposed a model for pseudodrusen formation involving altered local lipid processing and impaired clearance of lipid‐rich photoreceptor debris in the subretinal space, due to RPE dysfunction. The abundance of esterified cholesterol within conventional sub‐RPE drusen, however, suggests impaired lipid transport across the Bruch's membrane‐choriocapillary complex and implies systemic delivery of diet‐derived lipoproteins (Spaide et al. 2018; Curcio et al. 2024).

The exact mechanisms underlying AMD development remain elusive. In a report on the outcomes of the 2024 Ryan Initiative for Macular Research (RIMR) meeting, Miller et al. proposed that “AMD represents a heterogeneous collection of disease states arising from the interplay of several dimensions constituting AMD risk or pathogenesis: genetics, ancestry, retinal imaging findings, diet and environment, ageing, and outer retinal molecular and cellular pathways”. This concept underscores that AMD is not a single disease but a spectrum of conditions with diverse pathophysiology and clinical outcomes (Miller et al. 2025). Our current understanding of biological mechanisms implies a multitude of often interlinked pathways relevant to the outer retina dysmetabolism in AMD (Guymer and Campbell 2023; Fleckenstein et al. 2024). Existing research has consistently demonstrated complement system dysregulation (including at a genetic predisposition level) (Fritsche et al. 2016; Tzoumas et al. 2021; Hallam et al. 2017; Sharma et al. 2021; Chen et al. 2026b), inflammatory factors (Saini et al. 2017; Chen et al. 2026a), autophagic deficiency (Cerniauskas et al. 2020; Kaarniranta et al. 2023), oxidative stress (Senabouth et al. 2022; Chang et al. 2023), mitochondrial metabolism defects (Ebeling et al. 2021; Senabouth et al. 2022; Chen et al. 2026d), loss of lipid homeostasis (Pucchio et al. 2022; Curcio et al. 2011), RPE epithelial to mesenchymal transition (Sharma et al. 2021), intracellular cytoskeleton and extracellular matrix remodelling (Tarau et al. 2019; Navneet and Rohrer 2022), altered secretion (Kurzawa‐Akanbi et al. 2022; Flores‐Bellver et al. 2021), choroidal involvement (Manian et al. 2021; Biesemeier et al. 2014), and loss of retinal homeostasis through Müller glia dysfunction and reactive remodelling (Chen et al. 2026c) among others, as relevant to the outer retina dysfunction and degeneration.

The recognition of RPE cell morphological and transcriptional heterogeneity, and the existence of distinct RPE subpopulations with different susceptibilities to retinal disorders (Ortolan et al. 2022; Farjood et al. 2025), provide a framework for understanding the diversity of biological pathways involved in AMD. This functional diversity suggests that RPE subpopulation‐specific pathologies may exist and could account for differences in the progression and qualitative appearance of late AMD lesions (Mones and Biarnes 2018). Therefore, identifying biological pathways that are linked to specific AMD subtypes, or those universally relevant across all forms of AMD, may enable the development of precision therapies (Miller et al. 2025), and will likely inform diagnostic approaches. This prospect is increasingly attainable with advances in artificial intelligence (AI) and will undoubtedly contribute to the identification of biofluid (ocular and/or peripheral) markers and respective biological pathways for AMD diagnosis and personalised treatment selection (Pucchio et al. 2024, 2022).

Extracellular vesicles (EVs) have gained a remarkable interest in recent years as a novel liquid biopsy tool, due to encapsulating functional biological material into lipid bilayer‐enclosed vesicles, that reflect the parental cell's metabolic status (Kalluri and LeBleu 2020; Kurzawa‐Akanbi et al. 2024). The RPE cells secrete EVs in a polarised manner (Klingeborn et al. 2017) and apical/basal secretion patterns alongside EVs contents are altered in models of AMD‐RPE carrying a genetic predisposition (Kurzawa‐Akanbi et al. 2022) or exposed to environmental risk factors (Flores‐Bellver et al. 2021). Although ocular fluids such as the aqueous humour and the vitreous humour are sources of RPE and neuroretina‐derived EVs (Kalargyrou et al. 2022), EVs secreted toward the choroidal side can enter the systemic circulation, enabling retinal disease signatures to be detected in peripheral blood (Muraoka et al. 2022). Investigating blood EVs, therefore, provides a means to test the warranted hypothesis that retinal biomarkers are present in the bloodstream. Furthermore, the circulating EVs may shed light on the systemic EV delivery to the retina, a process that could theoretically contribute to the sub‐RPE drusen formation and AMD pathogenesis (Curcio et al. 2024).

In this study, EV‐enriched samples derived from plasmas of 30 AMD and 30 control individuals were subjected to proteomic, lipidomic and Raman spectroscopy analyses to define the peripheral molecular signatures and associated biological pathways in late‐stage AMD. Our findings reveal a distinct extracellular particle profile characterised by complement dysfunction, oxidative damage and lipid imbalance, corroborating previous studies on cellular mechanisms of AMD, and underscoring the relevance of the systemic extracellular particle signatures to late‐stage AMD pathology.

## 2 Materials and Methods

### 2.1 Study Cohort

Thirty plasma samples from AMD patients and 30 controls were derived from the University of Southampton, collected previously as part of ethically approved studies and with consent for use in other ethically approved studies on AMD. The research reported here received an independent favourable ethical opinion from the Proportionate Review Sub‐committee of the London—Riverside Research Ethics Committee (REC reference: 20/PR/0772). The demographic and selected clinical information per subject group is presented in Table 1 (see Table S1 for extended patient clinical information).

**TABLE 1:** Descriptive statistics for the study cohort.

	AMD cases	Controls
**No of individuals**	30	30
**GA**	13	0
**CNV**	5	0
**Mixed GA/CNV**	12	0
**Age Mean ± Standard deviation** ^a^	74.7 ± 10.6	70 ± 10.6
**Body Mass Index (BMI)** ± **Standard deviation** ^a^	27.8 ± 6	26.3 ± 3.6
**Gender: Female**	20	15
**Gender: Male**	10	15
**Current smoker**	3	8
**Past smoker**	12	21
***CFH* rs1061170 CC/CT/TT** ^b^	14/6/10	2/9/19
***HTRA1/ARMS2* rs10490924 TT/GT/GG** ^a^	5/14/11	4/6/20

Abbreviations: CNV, choroidal neovascularisation; GA, geographic atrophy.

^a^No statistically significant difference for age, BMI and *HTRA1/ ARMS2* rs10490924.

^b^ *p* < 0.01 for *CFH* rs1061170 (see results for details).

Plasma samples were prepared as described previously (Gibson et al. 2012). Plasma was obtained from fresh blood collected in lithium heparin tubes by centrifugation at 2600 rpm for 10 min. Plasma samples were stored at −80°C. They were transported to Newcastle University on dry ice and stored at −80°C until use.

### 2.2 EV‐Enriched Sample Isolation From Plasma

As we developed protocols, initially twenty plasma samples were defrosted on ice (10 AMD and 10 controls, see Table S1 for details). As platelet‐derived vesicle formation during the defrost was suspected and to preserve the integrity of the biofluid and EVs (Trummer et al. 2009; Noordin et al. 2018; Selleng and Greinacher 2021), the remaining 40 samples were defrosted rapidly at ambient temperature and immediately placed on ice (see Table S1 for case details). All samples were spun at 1500 × *g* at 4°C, followed by 3000 × *g* at 4°C twice, 10 min each step, each time the supernatant was subjected to the centrifugation, and pellet removed. Five hundred microliters of pre‐cleared plasma was loaded onto the qEV Original Gen2 70 nm size exclusion chromatography column (Izon Science). EV isolation was performed as per manufacturer's recommendations; 2.9 mL of void buffer volume was collected, followed by 1.6 mL of EV‐enriched fraction in freshly 0.22 µm‐filtered phosphate‐buffered saline (PBS, Sigma). The column was sanitised after each sample with 8.5 mL of 0.5 M NaOH (Sigma) and flushed with at least 30 mL of PBS, before loading the next plasma sample. EV‐enriched samples were concentrated to approximately 500 µL by ultrafiltration using Amicon Ultra 10 kDa regenerated cellulose units (Millipore). The samples were aliquoted into working volumes and stored at −80°C until use.

The distribution of samples used for proteomic and lipidomic analyses was balanced with respect to the plasma thawing protocol. For proteomics, the slow‐thaw cohort comprised 6 AMD and 6 control samples, while the rapid‐thaw cohort comprised 5 AMD and 5 control samples. For lipidomics, the slow‐thaw cohort comprised 9 AMD and 8 control samples, while the rapid‐thaw cohort comprised 9AMD and 10 control samples. Raman spectroscopy was performed in an independent cohort, for which all plasma samples were rapidly thawed at ambient temperature prior to processing (Table S1).

As a study limitation, the potential impact of plasma thawing protocol on the integrity of EV‐enriched samples was evaluated. Proteomic analysis showed a trend toward a greater and more consistent number of detected proteins in EV‐enriched samples derived from rapidly thawed plasma compared with samples derived from plasma thawed on ice; however, these differences did not reach statistical significance (Figure S4). The effect of thawing protocol on lipid detection was not assessed.

### 2.3 Transmission Electron Microscopy (TEM)

Five microliters of EV‐enriched samples were applied to carbon‐coated and glow‐discharged copper grids (Gilder Grids) for 2 min. Excess sample was removed gently with filter paper, following which the adsorbed sample was negatively stained with 2% aqueous uranyl acetate (Agar Scientific) for a few seconds. The grids were dried under a lamp before being examined on a Hitachi HT7800 transmission electron microscope using an Emsis Xarosa camera with Radius software.

### 2.4 Tunable Resistive Pulse Sensing (TRPS)

A qNano (Izon Science Ltd., New Zealand) was used to complete all the measurements for this study, detailed description of the set up can be found elsewhere (Blundell et al. 2015). A qNano uses data capturing software (Izon Control Suite v3.3) to record the particles as they traverse the pore. The lower fluid cell contained 80 µL of 1× PBS solution and the upper fluid cell contained 40 µL sample solution. After each measurement was taken, the nanopore was cleaned by first rinsing the upper fluid cell with background buffer before the buffer was removed and replaced multiple times. The qNano was operated with a positive bias, that is, the positive electrode in the lower fluid cell and the ground electrode in the upper fluid cell, so the particles traverse the pore toward the positive electrode unless otherwise stated. In these experiments, both a NP150 and NP200 nanopore was used to be able to analyse particles from 85 to 500 nm. Each pore was first characterised using the calibration particles so day‐to‐day differences could also be accounted for. Calibration particles were supplied by Izon Science.

### 2.5 EV‐Enriched Samples Protein Concentration Measurement and Western Blot

The EV‐enriched samples protein concentration was determined using Pierce Micro BCA Protein Assay Kit (Thermo Fisher Scientific). An aliquot of the particle suspension was lysed with Triton X‐100 (Sigma, 1% final concentration) and analysed according to the manufacturer's instructions. Alternatively, the samples were lysed with SDS (Sigma) at a final concentration of 0.1%, and the protein concentration was determined using a Qubit Protein Assay Kit (Thermo Fisher Scientific).

For Western blotting, 0.5–2.5 µg of extracellular particle proteins (without prior lysis) were combined with NuPAGE LDS sample buffer and NuPAGE sample reducing agent (reducing agent was not used for the analysis of CD63, CD9 and CD41). Samples were heated at 70°C for 10 min prior to electrophoresis in NuPAGE Bis‐Tris 4%–12% protein gels with NuPAGE MES SDS running buffer (all reagents from Thermo Fisher Scientific) and alongside the PageRuler Plus Prestained Protein Ladder (Thermo Fisher Scientific). The gels were transferred to PVDF membranes using the iBlot 2 transfer stacks and the iBlot 2 gel transfer device (Thermo Fisher Scientific). Post‐protein transfer membrane blocking was carried out using 5% dried skimmed milk solution in Tris Buffered Saline (Santa Cruz Biotechnology) with 0.1% Tween 20 (BioRad) (TBST). The membranes were incubated with primary antibodies overnight at 4°C with gentle agitation, followed by four washes (10, 20, 20, 10 min long), secondary HRP‐conjugated antibody incubation for 1 h at room temperature (see Table S2 for primary and secondary antibody list) and subsequent washes in TBST (as previously). Protein levels were detected using SuperSignal West Femto Maximum Sensitivity Substrate or SuperSignal West Pico PLUS chemiluminescent substrate (both Thermo Fisher Scientific) as per manufacturer's guidelines. Chemiluminescence imaging was performed with the Amersham Imager 600 (Cytiva, formerly GE Healthcare) and image analysis was carried out using the ImageQuant TL software v 8.2 (Cytiva). Western blots were performed at least three times per target. Densitometry data were analysed for normality with the Shapiro–Wilk test, followed by parametric unpaired *t* test with Welch's correction or non‐parametric Mann Whitney test to determine statistical significance, using GraphPad Prism v10.6.1 (892) software. Data were presented as Mean ± SEM with the statistical significance indicated on graphs as ns (not significant) or * *p* < 0.05, ** *p* < 0.01, **** *p* < 0.0001.

### 2.6 Proteomics and Data Analysis

EV‐enriched samples were lysed by boiling in 2% SDS buffer (2% (w/v) SDS, 0.5 mM EDTA, 50 mM HEPES, pH 8.0, 1× HALT phosphatase and protease inhibitor cocktail (Thermo Fisher Scientific)) for 4 min at 95°C, followed by sonication in a Bioruptor device (Diagenode) for 5 min with 30 s on‐and‐off cycles to ensure complete lysis and to shear any remnant DNA. Protein concentrations were determined by Micro BCA protein assay (Thermo Fisher Scientific) according to manufacturer instructions. Lysed protein samples were reduced with Tris‐(2‐carboxymethyl)‐phosphine hydrochloride (TCEP) at 10 mM final concentration and alkylated with 2‐chloroacetamide (CAA) at 40 mM final concentration. SP3 protein clean‐up and digestion was performed with five microgram protein per sample using Sera‐Mag SpeedBead carboxylate‐modified magnetic particles (E3 and E7; Cytiva) according to manufacturer instructions with slight modifications: (i) protein binding was initiated by adding 100% acetonitrile instead of ethanol; (ii) samples were incubated for 5 min to enable protein‐bead binding; (iii) one extra washing step with 100% acetonitrile was performed following the three washes with 80% (v/v) ethanol); and (iv) beads were reconstituted in 100 mM tetraethylammonium boride (TEAB) for overnight digestion. Peptide samples were dried by vacuum centrifugation in a SpeedVac and stored at −80°C until mass spectrometric analysis.

Peptide samples were analysed by data‐independent acquisition (DIA) mass spectrometry using an Orbitrap Exploris 480 (Thermo Fisher Scientific) mass spectrometer coupled to an Ultimate 3000 RSLCnano UHPLC system (Thermo Fisher Scientific) in injection triplicates. Dried peptides were re‐dissolved in 2% (v/v) acetonitrile (ACN), 0.05% (v/v) TFA in water and injected into the UHPLC system, where they were concentrated on a C18 PepMap100‐trapping column (0.3×5 mm, 5 µm; Thermo Fisher Scientific) and separated on an in‐house packed analytical C18 column (75 µm × 300 mm; Reprosil‐Pur 120C18‐AQ, 1.9 µm; Dr. Maisch GmbH) using a 103 min linear, stepped gradient of 9%–45% buffer B (80% (v/v) ACN, 0.1% (v/v) formic acid (FA) in water) at 300 nL/min (step 1: 9%–30% buffer B over 92 min; step 2: 30%–45% buffer B over 11 min). MS/MS data were acquired using 40 variable windows across a scan range of 350–1650 *m*/*z*. Precursor ion scans were acquired at a resolution of 120,000 in the orbitrap mass analyser. Precursor ions were fragmented by higher‐energy collisional dissociation (HCD) with a fixed collision energy of 30% and fragment ion scans were acquired with a resolution of 30,000.

Spectronaut (Biognosys; version 18.3) directDIA analysis of the generated MS/MS data was performed with default settings. Spectra were searched against a spectral library generated by Spectronaut from the UniProtKB human reference proteome (release 2023‐12‐06; 20,593 entries) for protein identification. The normalized protein group intensity matrix was exported for downstream analysis in Perseus (v2.0.6.0). Differential abundance analysis with normalised signal intensities was performed on log2‐transformed intensities, comparing the AMD group (A, *n* = 11) with the control group (C, *n* = 11) with three technical replicates per sample. Proteomics data were evaluated using permutation‐based false discovery rate (FDR) correction in Perseus; however, no proteins remained significant under this criterion. Consequently, differential protein abundance findings were assessed based on nominal *p* value and fold‐change thresholds and interpreted as exploratory. Multivariable adjustment for smoking status, sex, age, BMI, *CFH* genotype and *HTRA1/ARMS2* genotype was not performed due to the small sample size. Proteins were considered significantly differentially abundant if they exhibited an average fold change of ≥1.5 (log2 fold change ≥0.58 or ≤−0.58) and a *p* value ≤ 0.05 (−log_10_ (*p* value ≥ 1.3)). All detected and significantly enriched and depleted proteins are listed in Tables S3 and S4, respectively. Experimental reproducibility was confirmed by high Pearson correlation coefficients (*r* > 0.9) between technical replicates. Gene ontology (GO) enrichment was performed using ShinyGO (v0.85.1) with an FDR < 0.05. Protein–protein interaction (PPI) networks were generated via STRING (v12.0) using a high‐confidence interaction threshold (>0.7) and visualized in Cytoscape (v3.10.4).

### 2.7 Lipidomics and Data Analysis

Lipids were extracted from raw EV‐enriched samples with the addition of methanol/chloroform (2/1, v/v). The mixture was left to stand at 4°C for 1 h and then centrifuged to pellet the protein portion of the samples. Supernatants were transferred to glass tubes and evaporated to dryness under nitrogen gas. Samples were reconstituted in methanol containing 5 mM ammonium formate prior to LC‐MS analysis.

Samples (10 µL) were injected onto a Thermo Hypersil Gold C18 column (2.1 mm × 100 mm; 1.9 µm) maintained at 50°C on a Dionex UltiMate 3000 RSLC system. Mobile phase A consisted of water containing 10 mM ammonium formate and 0.1% (v/v) formic acid. Mobile phase B consisted of a 90/10 (v/v) mixture of isopropanol: acetonitrile containing 10 mM ammonium formate and 0.1% (v/v) formic acid. Lipids were eluted off the column using a gradient in which the percentage of mobile phase B was increased from 35% to 65% over 4 min, followed by 65%–100% over 15 min, with a hold for 2 min before re‐equilibration to the starting conditions over 6 min. Lipids were introduced to the Thermo Exactive Orbitrap mass spectrometer through a heated electrospray ionization (HESI) probe. Samples were analysed in both positive and negative ionisation mode over the mass‐to‐charge ratio (*m*/*z*) range of 250–2000 at a resolution of 100,000.

Raw LC‐MS data were processed with Progenesis QI v2.4 (Nonlinear Dynamics). Relative quantification was performed following normalisation to all detected compounds. Data were filtered based on ANOVA *p* value (<0.05), fold change (>1.2), and ANOVA FDR (<0.05). This workflow was applied to data acquired in both positive and negative ionisation modes. Significant features were identified by searching against the LIPID MAPS and HMDB databases using a mass tolerance of 5 ppm.

As the initial statistical analysis in Progenesis does not incorporate correction for multiple comparisons, a subsequent more stringent analysis was performed using two‐way ANOVA with Šídák's multiple comparisons test in GraphPad Prism (v10.6.0) on the significantly altered features. This additional step reduced the likelihood of false positives arising from multiple pairwise comparisons. The statistical significance of differences in the ratio of total sphingomyelin to total ceramide abundance in AMD versus controls was assessed using the Shapiro–Wilk test for normality, followed by the Mann–Whitney test. Data were presented as Mean ± SEM.

As a sensitivity analysis, we also created adjusted abundances of the identified significant features (in both positive and negative ion mode), adjusted for the effects of *CFH* rs1061170 genotype, *HTRA1/ARMS2* rs10490924 genotype and smoking (ever smoked). The adjusted lipid measurements were calculated as the residuals from a multiple linear regression analysis of the original lipid measurements on the covariates, with genotype considered as a 3‐level factor for both *CFH* and *HTRA1/ARMS2*. The adjusted measurements were taken forward to a two‐way ANOVA with Šídák's multiple comparisons test in GraphPad Prism (v11.0.2 (92)) software in the same way as the original raw measurements, to investigate whether the original findings were independent of, or potentially mediated through the effects of genotype and/or smoking.

### 2.8 Raman Spectroscopy

Sample preparation involved pipetting 5 µL of EV‐enriched samples onto glass slides wrapped in aluminium foil. They were allowed to air dry for 30 min at room temperature. Raman measurements were performed using a Renishaw inVia Qontor Raman spectrometer (Renishaw, UK), equipped with a 785 nm excitation laser, ×50 objective lens, and 1200 lines/mm grating.

Spectra were acquired across the 800–1800 cm^−^^1^ fingerprint region using approximately 65 mW laser power, with three accumulations of 5 s each. Five spectra were collected per sample and averaged to generate representative spectral profiles. Spectral preprocessing, including cosmic ray removal and baseline correction, was performed using WiRE software (Renishaw, UK).

Spectra were subsequently normalised using standard normal variate (SNV) transformation implemented in Python 3.7 to minimize baseline and scattering variation. A Savitzky–Golay second derivative transformation (window size 15, polynomial order 2) was applied to improve spectral resolution. Peak identification was performed using the “Peak Find” function in WiRE software. Overall spectral differences between AMD and control groups were assessed using PERMANOVA. Exploratory multivariate analysis using Partial Least Squares Discriminant Analysis (PLS‐DA) and Variable Importance in Projection (VIP) scoring was performed to identify spectral features contributing to group separation. Statistical analysis of selected Raman peaks was conducted using unpaired *t*‐tests in MetaboAnalyst 6.0, with significance defined as *p* < 0.05. Multivariable adjustment for smoking status, sex, age, BMI, *CFH* genotype and *HTRA1/ARMS2* genotype was not performed due to the small sample size.

## 3 Results

### 3.1 Study Cohort

Plasma samples from 30 AMD patients and 30 control individuals, previously collected at the University of Southampton, were utilised in this study. There was no statistically significant difference in the individuals’ age at collection for both groups, as determined by Shapiro–Wilk and Mann–Whitney tests (*p* = 0.0726, Table 1). Similarly, body mass index (BMI) did not differ significantly between patients and controls (*p* = 0.2292 from an unpaired *t*‐test with Welch's correction). The AMD cohort consisted of 13 patients with the diagnosis of geographic atrophy (GA), 5 patients with choroidal neovascularisation (CVN) and 12 patients with GA in one eye and CNV in the other eye (mixed GA/CNV phenotype) (Tables 1 and S1), representing the spectrum of phenotypes associated with late‐stage AMD. There was a significant overrepresentation of the *CFH* rs1061170 high‐risk CC variant among AMD patients (47% of AMD cases, Fisher's exact test, *p* = 0.0013). No statistically significant overrepresentation of the *HTRA1/ARMS2* rs10490924 high‐risk TT genotype was observed in this cohort; however, AMD patients exhibited a higher percentage of GT genotype carriers compared to controls (Fisher's exact test, overall *p* = 0.0622) (Tables 1 and S1).

**FIGURE 1:**
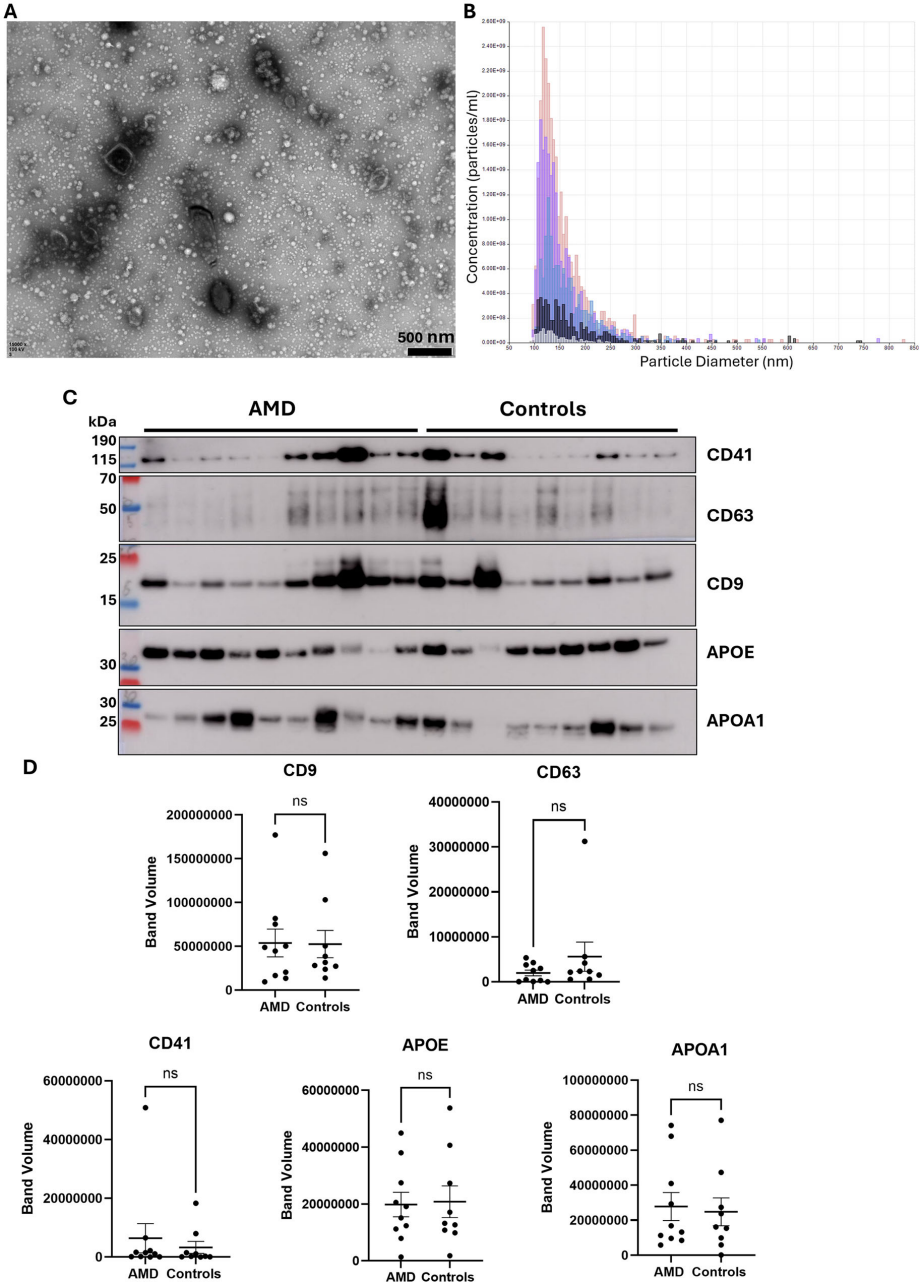
**Plasma EV isolates characterisation**. (A) Transmission electron microscopy image of negatively stained plasma EVs preparations. Vesicles with classic cup‐shaped morphology characteristic for EVs are present, alongside other particles, likely lipoproteins, commonly co‐isolated with EVs. (B) Representative image of plasma extracellular particles size distribution as determined by Tunable Resistive Pulse Sensing analysis. (C) Representative Western blot analysis of the abundance of EV markers CD9 and CD63, alongside co‐isolates: platelet‐derived particles indicated by CD41 presence and lipoproteins APOE and APOA1. The analysis was performed for *n* = 29 AMD and 29 controls. (D) Western blot image densitometry analysis of at least 3 independent replicates was performed in ImageQuant TL 8.2. and analysed statistically in GraphPad Prism. *n* = 10 AMD and 9 controls per replicate; ns‐ not significant.

Plasma samples were processed for EV isolation using differential centrifugation and size exclusion chromatography. TEM of negatively stained EV‐enriched isolates demonstrated the presence of extracellular particles (Welsh et al. 2024), including vesicles with collapsed morphology (expected for EVs) (Figure 1A). Tunable resistive pulse sensing (TRPS) analysis of 20 samples demonstrated a mean particle size of 160 nm (standard deviation 14.7 nm) with an average mode of 122.6 nm (standard deviation 7 nm) (Figure 1B). Because the blood collection and plasma preparation protocols were not optimised to minimise EV co‐isolates (blood samples were collected as part of earlier studies at the University of Southampton), we considered that the isolates likely contained common confounders such as lipoproteins and platelet‐derived vesicles (Nieuwland et al. 2022). We therefore assessed the isolates for the abundance of EV markers CD9 and CD63, the platelet marker CD41, and the lipoprotein components APOE and APOA1 by Western blotting. High abundance of CD9 and presence of CD63 tetraspanins in the isolates validated successful derivation of EVs from plasma (Figures 1C and S1) (Welsh et al. 2024). Variable levels of CD41, APOE and APOA1 were also detected, with no statistically significant differences in abundance between AMD and control samples, indicating some degree of contamination with non‐EV particles (Figure 1D). We therefore refer to the isolates as “EV‐enriched samples” or “extracellular particle samples”, in accordance with the MISEV2023 guidelines (Welsh et al. 2024).

### 3.2 Global Proteomics of Plasma EV‐Enriched Samples Underscores Immune, Redox and Proteolysis Dysregulation in AMD

**FIGURE 2:**
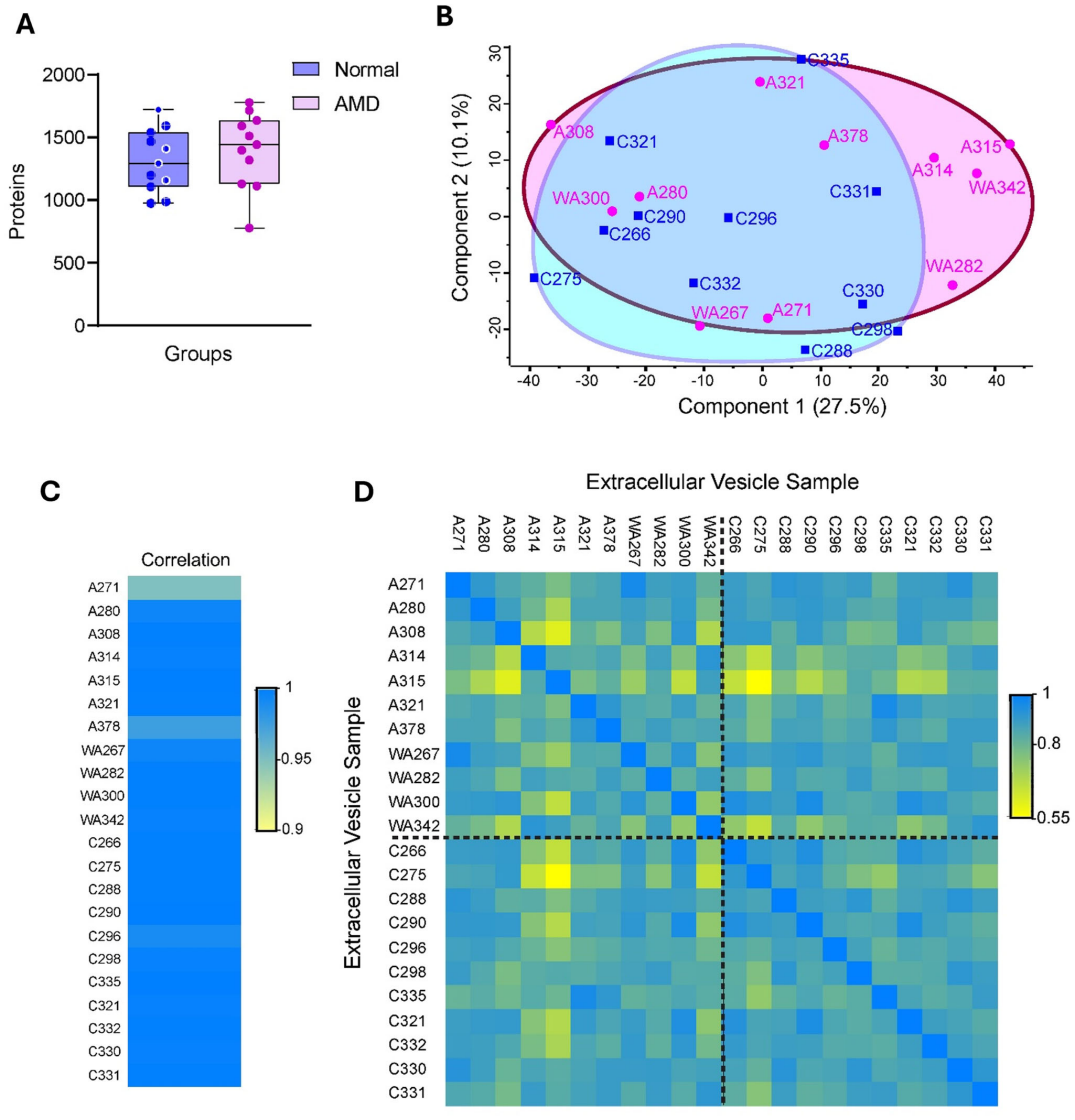
**AMD and control plasma EV‐enriched samples global proteomics**. (A) Box plots showing the number of identified proteins from EV‐enriched samples from unaffected controls (*n* = 11) and AMD patients (*n* = 11). The centre line indicates the median and the error bars standard deviation. (B) Principal component analysis of all samples. “C” in front of a number indicates a control normal individual, “A” in front of a number indicates dry AMD/geographic atrophy, “WA” in front of a number indicates wet AMD/choroidal neovascularisation. (C, D) Plots showing quantitative reproducibility between measured replicates (C) and the correlations between analysed samples (D).

**FIGURE 3:**
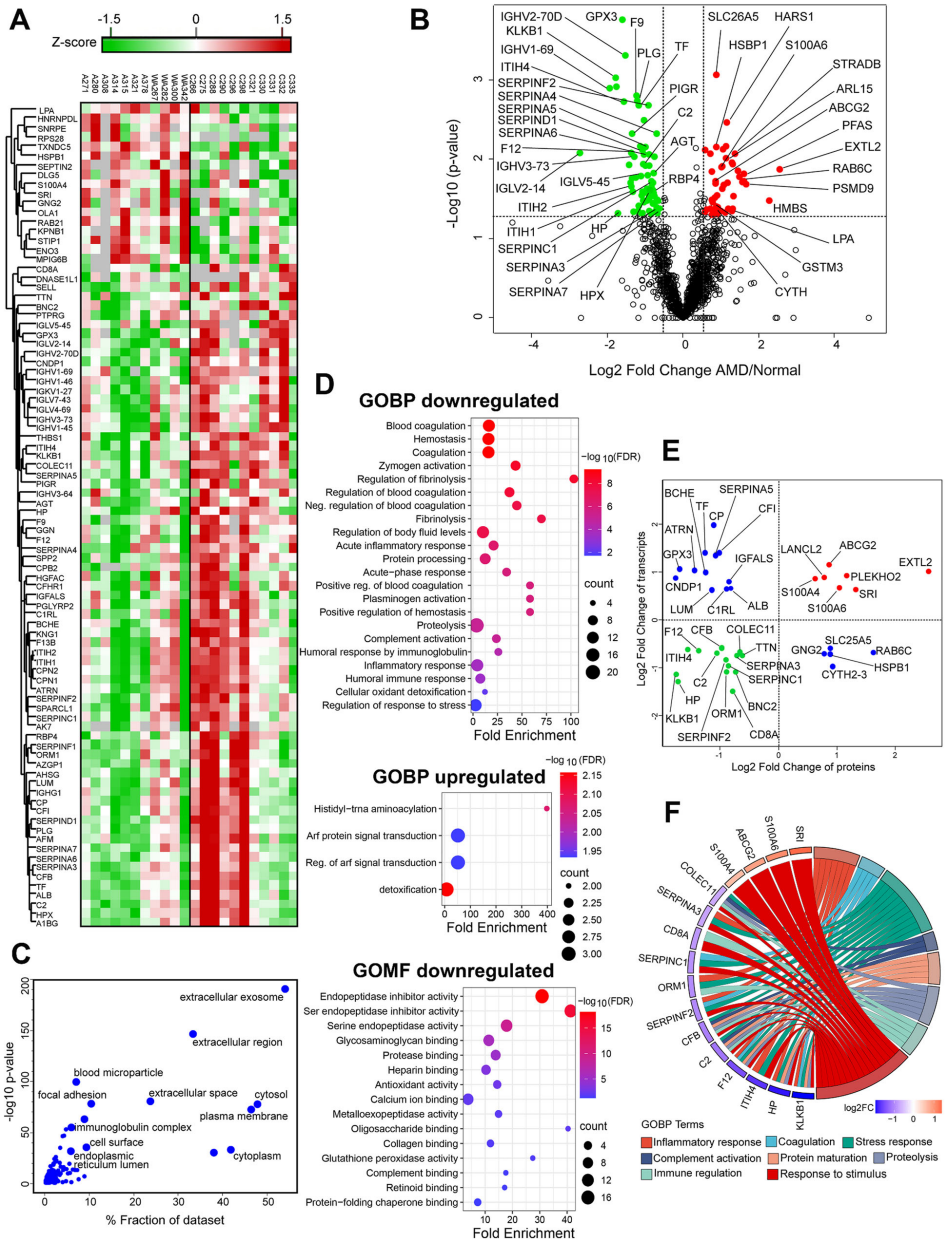
**Differentially expressed plasma extracellular particle proteins in AMD versus controls**. (A) Non‐supervised hierarchical clustering of significantly changed proteins (cutoff 1.5‐fold change/ log2 0.58), which were detected in at least 70% of samples (see **Figure** S2 for the full heatmap with all the detected proteins). (B) Volcano plot comparing *p* values and log2(fold change) values for plasma EV‐enriched protein abundance between AMD and control normal individuals. The most significantly upregulated and downregulated proteins are coloured in red and green, respectively. (C) Enrichment of extracellular vesicle proteins in the identified proteome. Gene Ontology Cellular Component (GOCC) enrichment analysis was performed using DAVID and the identified terms were plotted using the Perseus software. (D) Gene Ontology Biological Process (GOBP) and GO Molecular Function (GOMF) analyses of the differentially expressed proteins in AMD versus control plasma EV‐enriched samples (analysed and visualised using the ShinyGO version 0.85.1). Pathways and processes overrepresented among the downregulated and upregulated proteins in AMD versus controls are shown. (E) Correlation between significantly altered AMD EV‐enriched proteins and differentially expressed transcripts in the RPE‐choroid and retina from AMD donor patients (Newman et al. 2012). (F) GOBP enrichment analysis of the significantly altered genes from E that showed a positive correlation between protein and transcript level changes.

Label‐free proteomic analysis was performed on a subset of AMD and control plasma‐derived EV‐enriched samples (11 AMD: 7 GA and 4 CNV phenotype, and 11 controls), yielding >700 identified proteins per sample (Figure 2A). Principal Component Analysis (PCA) showed substantial overlap between the cohorts (Figure 2B). Quantitative reproducibility was confirmed by high Pearson correlation coefficients (*r* > 0.9) between measured replicates (Figure 2C). Control samples exhibited good correlation, whereas greater variability was observed among AMD samples and between AMD and control cases (Figure 2D). LC‐MS/MS data analysis identified 122 proteins differentially expressed (DE) between the AMD and control cohorts at an average fold change of ≥1.5 (*p* value ≤ 0.05) (Tables S3 and S4 and Figure S2). Among these, 48 proteins were statistically significantly enriched in AMD versus control samples, and 74 proteins were depleted in the AMD group. Figure 3A presents non‐supervised hierarchical clustering of DE proteins detected in at least 70% of EV‐enriched samples (88 proteins; see Figure S2 for the full heatmap with all DE proteins).

The most significantly altered proteins are presented in a volcano plot depicting *p* values and log2(fold change) of plasma extracellular particle protein abundance between AMD samples and controls (Figure 3B). Glutathione peroxidase (GPX3), a detoxifying enzyme that protects cells from oxidative damage by catalysing the reduction of hydrogen peroxide, lipid hydroperoxides and organic hydroperoxide, to water using glutathione (ENZYME entry: EC:1.11.1.9) (Brigelius‐Flohe and Maiorino 2013), was the most significantly depleted protein in AMD EV‐enriched samples. Other proteins with reduced abundance in AMD, including haptoglobin (HP), ceruloplasmin (CP), serotransferrin (TF) and hemopexin (HPX), are known to prevent oxidative stress through their vital roles in iron metabolism (Theilgaard‐Monch et al. 2006; Hellman and Gitlin 2002; Gomme et al. 2005; Tolosano and Altruda 2002). Notably, a large number of immune‐related proteins were significantly reduced in AMD plasma EV‐enriched samples, including plasma kallikrein (KLKB1, a potential therapeutic target in AMD), specific immunoglobulin chains, haptoglobin, coagulation factors, serpin proteins (serine protease inhibitors), complement proteins (C2, CFB, CFI, C1RL and CFHR1), T‐cell surface glycoprotein CD8 alpha chain, collectin 11 and attractin (Motta et al. 2023; Bouton et al. 2023; De et al. 2023; Selman and Hansen 2012; Addison et al. 2005; Duke‐Cohan et al. 1998). Oxidative stress and inflammation are among the most widely debated factors in retinal pathophysiology in AMD, hypothesised to arise from intrinsic drivers (e.g., high metabolic activity of the retina and genetic predisposition, including polymorphisms in complement genes) and/or extrinsic factors (e.g., smoking and high‐fat diet) (Datta et al. 2017), underscoring the importance of the present findings.

Conversely, multiple proteins involved in protein homeostasis were upregulated in AMD plasma EV‐enriched samples compared to controls (Figure 3B and Table S4). These included components of the ubiquitin‐proteasome system (e.g., 26S proteasome non‐ATPase regulatory subunit 9 (PSMD9), ubiquitin‐fold modifier‐conjugating enzyme 1 (UFC1) and E3 ubiquitin‐protein ligase RNF8), alongside stress response proteins associated with oxidative and endoplasmic reticulum stress, for example, heat shock protein beta‐1 (HSPB1), stress‐induced‐phosphoprotein 1 (STIP1), thioredoxin domain‐containing protein 5 (TXNDC5), glutathione S‐transferase Mu 3 (GSTM3), and copper chaperone for superoxide dismutase (CCS). These findings align with our current understanding of cellular responses to oxidative stress (Reichmann et al. 2018).

The Gene Ontology (GO) Cellular Component analysis demonstrated a significant enrichment of the identified proteome in “extracellular exosome” proteins, further validating the presence of extracellular vesicles in plasma extracellular particle sample preparations (Figure 3C, Tables S5 and S6). GO Biological Process (GOBP) term analysis of differentially expressed proteins between AMD and controls showed clustering of the downregulated proteins in AMD into pathways predominantly related to the coagulation and complement system (Figure 3D and Tables S8). These included marked downregulation of Factor IX, Factor XII, KLKB1, plasminogen (PLG), SERPINF2 and C2. Despite evidence of systemic complement activation in AMD (Scholl et al. 2008), the observed reduced levels of complement components within circulating particles from AMD plasma samples suggest disease‐associated changes in complement cargo loading and/or increased complement consumption outside the secreted particle compartment. In contrast, only a few upregulated proteins were enriched into pathways related to histidyl‐tRNA aminoacylation, Arf signal transduction and detoxification (Figure 3D and Table S7). The GO Molecular Function (GOMF) analysis of downregulated proteins showed predominant enrichment of proteins with endopeptidase inhibitor activity, including serpin proteins (Figure 3D and Table S9).

Overall, the proteomic analysis of plasma‐derived EV‐enriched samples indicated changes in immune function and blood coagulation factors, alongside abnormalities in antioxidant processes and protease regulation, suggestive of chronic stress in AMD. These results not only corroborate our current understanding of AMD pathophysiology but also advance our knowledge of the pathways that could have a more universal role in the AMD spectrum.

### 3.3 Analysis of the Proteome of AMD EV‐Enriched Samples Reveals Downregulation of Potential AMD Biomarkers

STRING analysis of protein–protein interactions (PPI) between the significantly upregulated and downregulated proteins revealed a strong interaction network confined exclusively to the downregulated proteins, indicating important functional links among these AMD plasma extracellular particle proteins (Figure S3A). Moreover, proteins of the most enriched functional pathways, including blood coagulation, complement cascades and the endopeptidase inhibitor pathways, showed high PPI enrichment scores (Figure S3B). A number of these proteins have been implicated as potential AMD biomarker candidates in previous studies (Sideri et al. 2025). These include serpin protease inhibitors, SERPINA4 (kallistatin), SERPINA5, SERPINA7, SERPINF1 (pigment epithelium‐derived factor; PEDF), ITIH2 (Inter‐alpha‐trypsin inhibitor heavy chain 2), ITIH1, complement proteins C1RL, CFHR1, CFI, and the retinol binding protein RBP4 (Sideri et al. 2025; Rinsky et al. 2021; Winiarczyk et al. 2024). PEDF is a potent inhibitor of angiogenesis deficient in the vitreous of AMD patients (Holekamp et al. 2002). Kallistatin, an inhibitor of kallikrein (KLKB1), is known to regulate angiogenesis and oxidative stress (Chao et al. 2018). ITIH2, which exhibits high inhibitory activity against trypsin, has been proposed as a risk factor for AMD progression through extracellular matrix remodelling (Qu et al. 2019). Consistently, searching for disease‐associated genes among the significantly regulated proteins identified AMD‐related genes from the complement system, including C2, CFI, CFB, CFHR1 and F13B (Figure S3B). These genes have been implicated as important genetic risk factors and molecular drivers of AMD (Armento et al. 2021). In addition, GPX3, the most significantly downregulated protein in our proteomic dataset, was previously detected as an upregulated protein in the vitreous humour of AMD patients (Koss et al. 2014). In contrast to the downregulated proteins, no high‐confidence PPI network was observed among the significantly upregulated proteins.

Next, we compared our AMD EV‐enriched proteomic dataset with the results of a previous transcriptomic analysis of RPE‐choroid and retina tissue samples from AMD human donor eyes (Newman et al. 2012). Strikingly, a total of 38 significantly altered proteins from our dataset were found to be differentially expressed at the transcript level in human RPE‐choroid and retina samples (Figure 3E), of which 21 proteins showed a positive correlation in abundance change. The GOBP analysis of these proteins revealed significant enrichment of inflammatory and stress responses, as well as coagulation and complement activation (Figure 3F). These findings are in strong agreement with our previous results and demonstrate the utility of this proteomic dataset for biomarker discovery.

**FIGURE 4:**
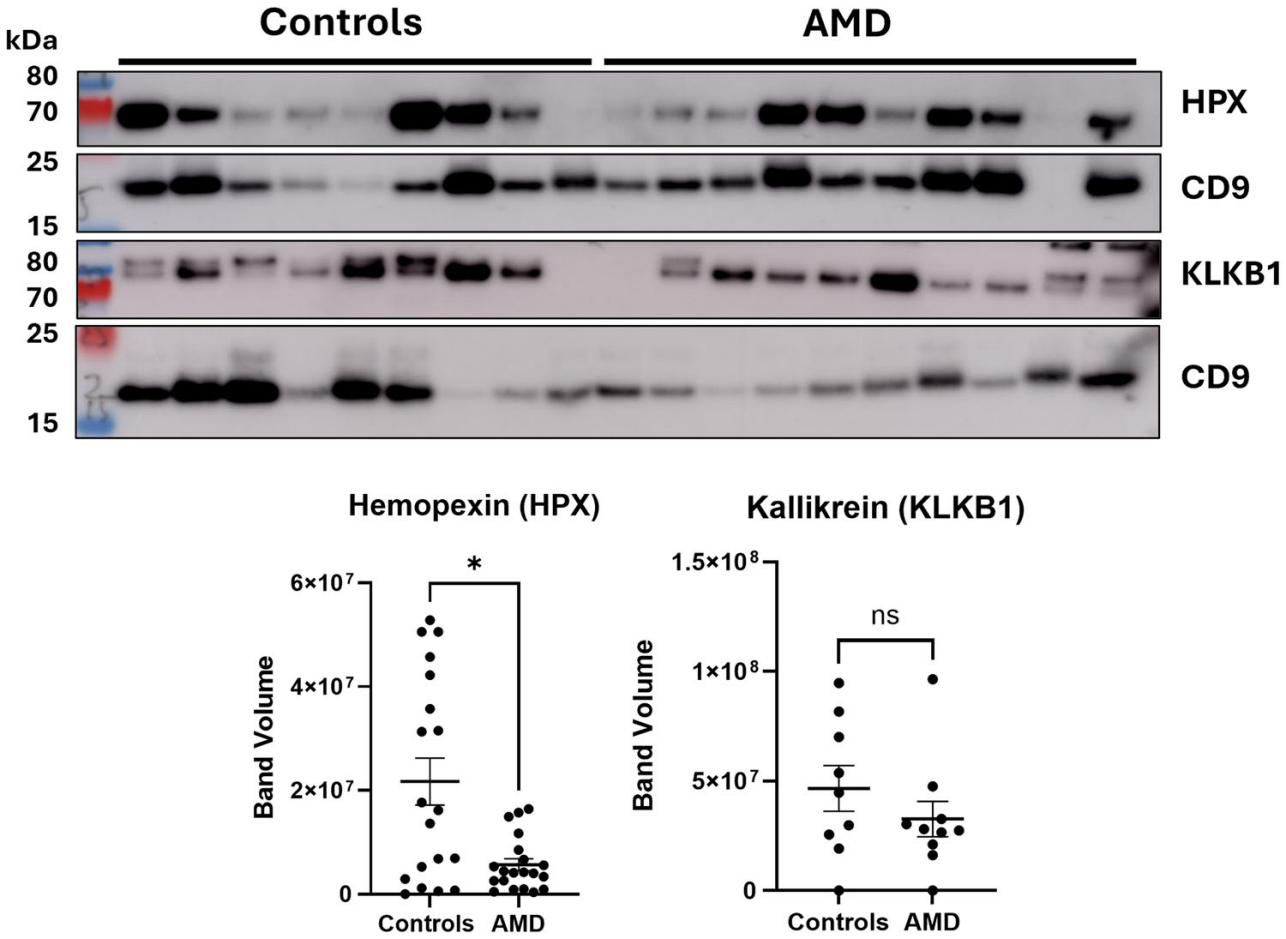
**Selected protein target validation in an expanded cohort of AMD and control plasma EV‐enriched samples**. Western blot‐based evaluation of hemopexin (HPX) and kallikrein (KLKB1) protein levels in EV‐enriched samples from AMD and control plasmas. CD9 protein expression shown to demonstrate EV marker abundance in the isolates. The same protein amounts as determined by the Qubit Protein Assay were loaded per lane. Statistical analysis of densitometry data from Western blot images (*n* = 9‐19 controls, 10–20 AMD), * indicates *p* < 0.05 from Mann–Whitney test. Mean with SEM is shown.

To provide independent validation of our findings, we screened specific targets identified as significantly changed in the mass spectrometry proteomics analysis using expanded cohorts of AMD and control plasma‐derived EV‐enriched samples. Hemopexin (HPX), which was also found to be differentially expressed in iPSC‐derived AMD RPE EVs in our previous study (Kurzawa‐Akanbi et al. 2022), was significantly reduced in AMD plasma‐derived EV‐enriched samples compared to controls, as determined by Western blotting (Figure 4). KLKB1 protein levels, functionally linked to wet AMD through vascular inflammation and abnormal blood vessel growth, and a potential therapeutic target (Hachana et al. 2020; Othman et al. 2021), were generally lower in AMD samples, although this difference did not reach statistical significance (Figure 4).

In summary, our proteomics study of AMD and control plasma‐derived EV‐enriched samples identified changes in proteins previously proposed as AMD biomarkers, underscoring biological processes such as complement system dysfunction and oxidative imbalance, that are likely relevant to the disease pathobiology (Sideri et al. 2025). The detection of those changes in EV‐enriched particles adds a new dimension to the AMD biomarker discovery and understanding of disease mechanisms.

### 3.4 Plasma EV‐Enriched Samples Lipidomics Indicates Dysregulated Sphingolipid Metabolism in AMD

**FIGURE 5:**
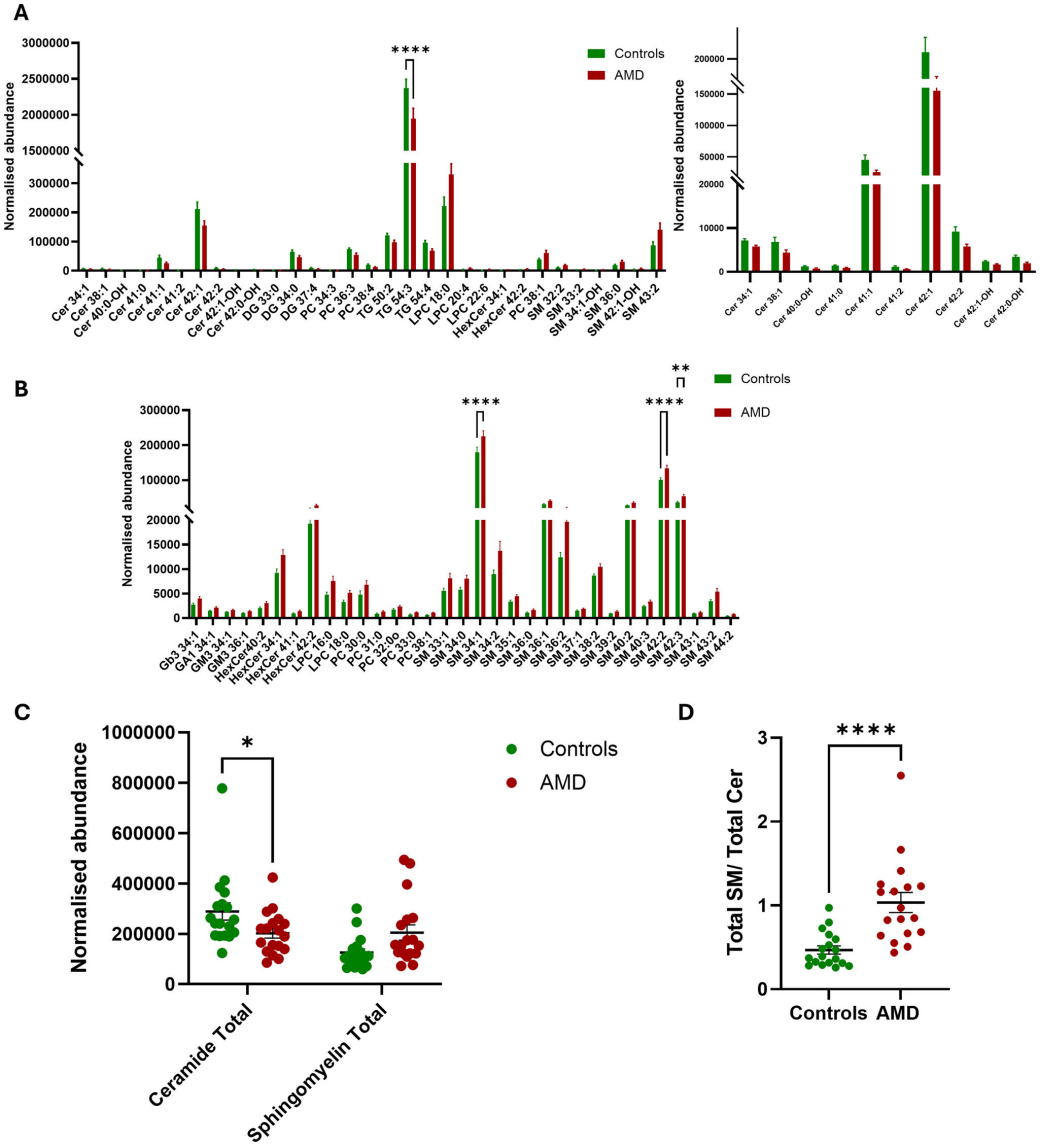
**AMD and control plasma EV‐enriched samples global lipidomics**. (A) Differentially abundant lipid features identified in positive ion mode by Progenesis QI v2.4 software. Normalised abundance of the lipid features was compared across groups by two‐way ANOVA with Šídák's multiple comparisons correction. **** Indicates *p *< 0.0001. The graph on the right presents a selection of data included in the full graph on the left, to visualise changes in ceramides. (B) Differentially abundant lipid features identified in negative ion mode by Progenesis QI v2.4 software. Normalised abundance of the lipid features was compared across groups by two‐way ANOVA with Šídák's multiple comparisons correction. ** Indicates *p* = 0.0091 and **** indicates *p* < 0.0001. (C) Normalised abundance of total ceramide and sphingomyelin in plasma EV‐enriched samples from AMD patients and controls. Data analysed using two‐way ANOVA with Šídák's multiple comparisons test. * Indicates *p* = 0.04. (D) Comparison of the ratio of total sphingomyelin to total ceramide abundance in AMD versus controls. Data analysed with the Shapiro–Wilk test for normality, followed by the Mann–Whitney test. **** indicates *p* < 0.0001. Mean with SEM shown. Cer, Ceramide; DG, Diacylglycerol; PC, Phosphatidylcholine; TG, Triacylglycerol; LPC, Lysophosphatidylcholine; HexCer, Hexosylceramide; SM, Sphingomyelin; Gb3, Globotriaosylceramide; GA1, Ganglioside GA1; GM3, Ganglioside GM3.

Differential analysis of the total lipid content of AMD and control plasma EV‐enriched samples identified several lipid species among glycerophospholipids, sphingolipids and glycerolipids that were altered in expression between the two groups (Tables S10 and S11). These included various molecular species of phosphatidylcholine, lysophosphatidylcholine, ceramide, hexosylceramide, sphingomyelin, globotriaosylceramide, ganglioside GA1 and GM3, diacylglycerol and triacylglycerol. Subsequent more stringent statistical analysis of the identified lipids using two‐way ANOVA with Šídák's multiple comparisons test showed a statistically significant decrease in one triacylglycerol species, alongside increases in several sphingomyelin species in AMD compared with controls (Figure 5A, B). Given the significant overrepresentation of the *CFH* rs1061170 genotype in the AMD cohort, as well as the imbalance for *HTRA1/ ARMS2* rs10490924 and smoking status, we have explored whether the observed statistically significant changes in specific lipid species are independent of, or, alternatively, potentially mediated through the effects of genotype and/or smoking. We therefore created adjusted abundances of the identified significant features (in both positive and negative ion mode), adjusted for the effects of *CFH* rs1061170 genotype, *HTRA1/ARMS2* rs10490924 genotype and smoking (ever smoked). Consistent with non‐adjusted analysis, statistically significant differences were found for triacylglycerol TG 54:3, and two sphingomyelin species, SM 34:1 and SM 42:2 (Figure S5). Sphingomyelin SM 42:3, was, however, no longer statistically significantly changed between AMD and controls upon adjustment for genotype and smoking status. This suggests that the statistically significant changes in the triacylglycerol species TG 54:3, and sphingomyelins SM 34:1 and SM 42:2 in AMD versus control plasma EV‐enriched samples are likely independent of the effects of *CFH* rs1061170 genotype, *HTRA1/ARMS2* rs10490924 genotype or smoking.

Since various ceramides showed a consistent trend toward decreased levels in AMD (Figure 5A), we calculated and compared the total amounts of identified ceramides between AMD and controls. This analysis demonstrated a statistically significant downregulation of pooled ceramides in AMD relative to controls (Figure 5C). Given a direct metabolic relationship between ceramide and sphingomyelin through interconversion (Hannun and Obeid 2011), we calculated and compared the ratio of total sphingomyelin to total ceramide between AMD and controls. This revealed a statistically significant increase in the total sphingomyelin to ceramide ratio in AMD (Figure 5D). Due to the interconnected nature of these metabolic pathways, these results may indicate decreased ceramide production and/or increased rate of sphingomyelin synthesis or reduced sphingomyelin breakdown to ceramide (Hannun and Obeid 2011), processes important for maintaining cellular ceramide homeostasis.

In summary, these data demonstrate disturbances in lipid metabolism associated with AMD, reflected by lipid changes in circulating particles. The prominent abnormalities in levels of sphingomyelin and ceramide are likely indicators of altered metabolic processes. Moreover, owing to the bioactive roles of these lipids, such changes may have broader effects, with a potential contribution to abnormal cholesterol homeostasis, protein metabolism via autophagic dysfunction and cellular stress, including oxidative stress (Gabandé‐Rodríguez et al. 2014; Kikas et al. 2018; Carsana et al. 2022). Importantly, these results parallel our previous observations linking abnormal sphingolipid levels with the high‐risk AMD RPE cells and their EVs (Kurzawa‐Akanbi et al. 2022) and the associations of candidate genes involving lipid metabolism (Fritsche et al. 2016; Jaskoll et al. 2025).

### 3.5 Raman Spectroscopy of Plasma EV‐Enriched Samples Validates an AMD Systemic Signature of Oxidative Stress and Dysregulated Lipid Metabolism

**FIGURE 6:**
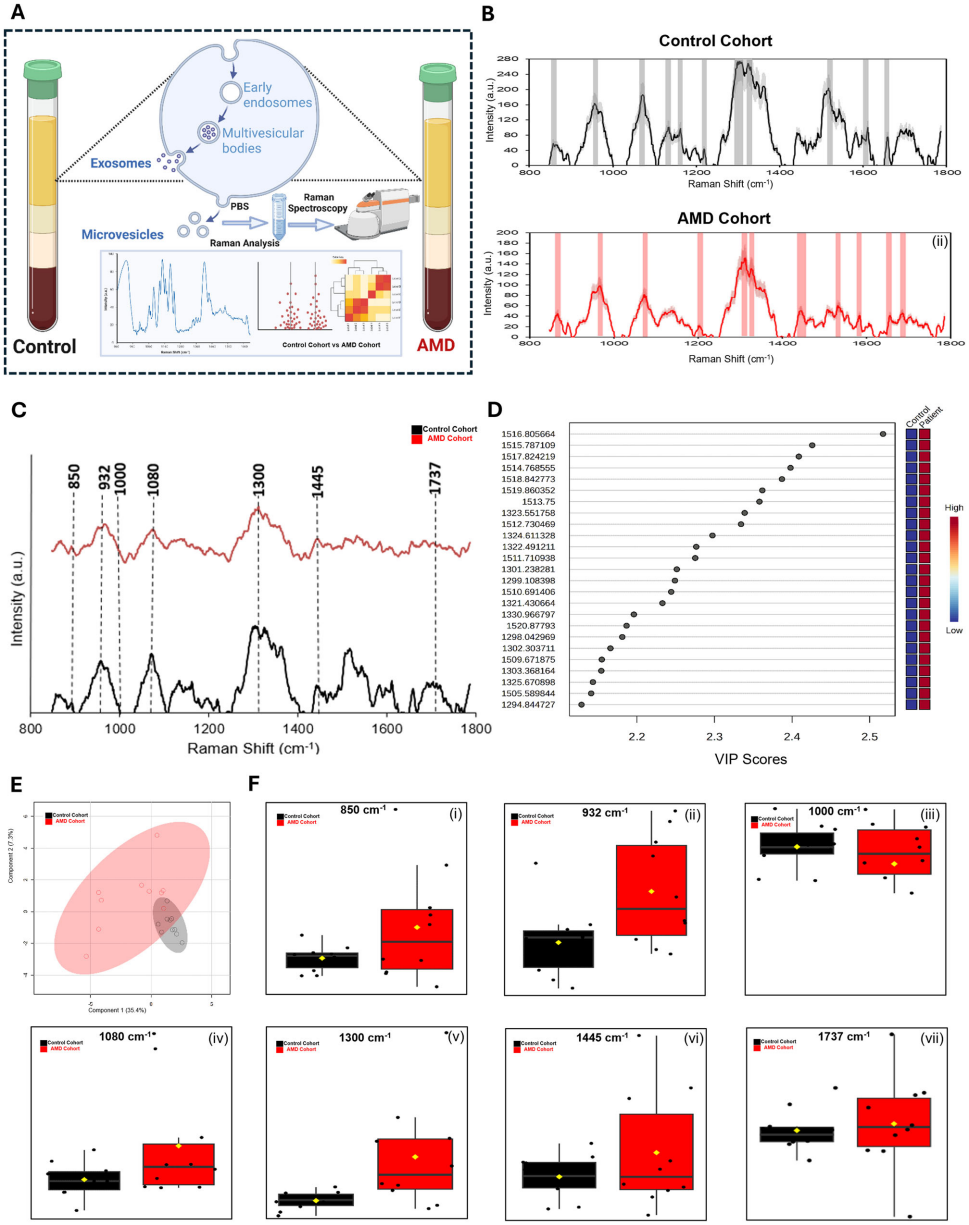
**Raman spectral analysis of plasma‐derived EV‐enriched samples from control and AMD cohorts**. (A) Experimental schematic outlining the derivation of EV‐enriched samples from plasma, with data collection performed using Raman spectroscopy (generated with BioRender). (B) Spectral barcodes displaying the average Raman spectra with standard deviation for (i) control (*n* = 10) and (ii) AMD (*n* = 10) samples, highlighting dominant peaks. (C) Key differentiating peaks between control and AMD cohorts: 850, 932, 1000, 1080, 1300, 1445, and 1737 cm^−^^1^. (D) VIP plot showing the top 25 significant peaks (VIP > 2) with an accompanying heatmap illustrating group clustering. (E) PLS‐DA score plot demonstrating partial overlap and increased dispersion within the AMD samples. (F) Box plots for key peaks indicating significant differences confirmed by unpaired *t*‐tests: (i–vii) *p* < 0.01 ** (932 cm^−^^1^, 1080 cm^−^^1^, 1300 cm^−^^1^, and 1445 cm^−^^1^), and *p* < 0.05 * (850 cm^−^^1^, 1000 cm^−^^1^, and 1737 cm^−^^1^). The centre line indicates the median.

Raman spectroscopy provides detailed chemical and structural characterisation of various samples, commonly referred to as a chemical fingerprint (Butler et al. 2016). Recent advances in Raman spectroscopy applications in profiling biological specimens, including EVs (Welsh et al. 2024), show its powerful utility in differential sample characterisation with diagnostic potential (O'Toole et al. 2024; Penders et al. 2021). We have therefore used label‐free Raman spectroscopy to analyse an independent cohort of 10 AMD and 10 control plasma EV‐enriched preparations (Figure 6A). Averaged spectral profiles and derived barcodes enabled comparative biochemical profiling, revealing distinct molecular signatures associated with AMD pathology. Spectral barcodes derived from averaged control and AMD samples (*n* = 10 each group), along with their standard deviations, are shown in Figure 6B.

Comparative spectral analysis revealed several Raman shifts with statistically significant intergroup differences. Notably, the peak at 850 cm^−^^1^ (tyrosine/protein side chains) exhibited altered intensities in the AMD cohort, suggesting protein compositional changes or oxidative modifications; the peak at 932 cm^−^^1^ (C–C skeletal stretch, α‐helical proteins) showed a robust difference, indicating disruption in protein secondary structure, a feature linked to cellular stress; the 1000 cm^−^^1^ peak (phenylalanine ring breathing) reflected differences potentially tied to amino acid metabolism; the 1080 cm^−^^1^ peak (PO_2_^−^ symmetric stretching, nucleic acids) was significantly altered, implying nucleic acid degradation or fragmentation consistent with oxidative stress in AMD; peaks at 1300 cm^−^^1^ and 1445 cm^−^^1^ (CH_2_/CH_3_ vibrations, lipids and proteins) suggested dysregulated lipid metabolism and disrupted protein conformation. The 1737 cm^−^^1^ peak (C = O stretching, lipid esters) showed increased intensity, potentially indicative of lipid peroxidation or accumulation of esterified compounds (Figure 6C) (Socrates 2001; Talari et al. 2015).

Multivariate analysis was performed to assess the discriminative capacity of these spectral alterations. Variable importance in projection (VIP) scores derived from partial least squares discriminant analysis (PLS‐DA) identified the top 25 spectral features (VIP > 2) contributing most to group separation. A corresponding heatmap demonstrated consistent intra‐group clustering and inter‐group separation. Although some overlap was observed, particularly among AMD samples, the increased spectral dispersion within the AMD cohort reflects underlying biochemical heterogeneity characteristic of the disease's multifactorial nature (Figure 6D, E). Partial Least Squares Discriminant Analysis (PLS‐DA) and Variable Importance in Projection (VIP) analyses were used as exploratory multivariate approaches to examine patterns of spectral variation and identify Raman features contributing to group separation. Although separation trends between AMD and control samples were observed, some overlap remained between groups, reflecting the biological heterogeneity of AMD and indicating that these analyses should be interpreted as hypothesis‐generating rather than predictive classification models.

To validate key spectral biomarkers, individual Raman shifts were analysed statistically using unpaired *t*‐tests and visualised with box plots (Figure 6F). Peaks at 932cm^−^^1^, 1080cm^−^^1^, 1300cm^−^^1^ and 1445cm^−^^1^ showed highly significant differences (p** < 0.01), while those at 850cm^−^^1^, 1000 cm^−^^1^ and 1737 cm^−^^1^ reached significance at p* < 0.05, supporting the robustness of the findings.

## 4 Discussion

The growing understanding of clinical aspects, risk factors and molecular cell biology of AMD has revealed the complexity and multidimensional nature of this condition. These observations underlie the recently proposed conceptual model of AMD as a spectrum or “cloud” of phenotypes. These phenotypes could be delineated by correlating risk and lifestyle factors with disease mechanisms, clinical presentation and outcomes, serving as independent dimensions that define the condition (Miller et al. 2025). Deciphering the interactions among risk factors, mechanisms, and outcomes has the potential to enable precision medicine by discovering highly predictive progression rates, selecting the most subtype‐appropriate therapies based on underlying biology, and accelerating drug development. Although defining distinct AMD subtypes will undoubtedly require a specific task force and a population‐based approach to track AMD progression and build a comprehensive dataset (Miller et al. 2025), our own studies of AMD pathobiology suggest that certain mechanisms may be dominant, and potentially universal across all forms of AMD. We hypothesise that, as AMD has features of a neurodegenerative condition, as well as a vascular disease, through shared risk factors and biological mechanisms identified by us and others (see below), a deeper exploration of the interconnectedness between these two pathologies will lead us to better‐defined prevention and treatment strategies.

In this study, we integrated multi‐omics with Raman spectroscopy to identify the systemic signature of AMD plasma extracellular particles. Our ‐omics analyses demonstrated dysregulation of the immune system, redox homeostasis, and protein and lipid metabolism, complemented by Raman spectroscopic evidence of biochemical spectral alterations associated with oxidative stress and lipid dysregulation. Further studies in larger cohorts will help determine the reproducibility and broader applicability of these spectral and ‐omics signatures.

Notably, the current findings align with our published in vitro studies using a patient‐specific model of AMD RPE carrying high‐risk polymorphisms in the complement factor H (*CFH*) gene, which unravelled the importance of RPE EV‐mediated signalling in the retinal niche (Kurzawa‐Akanbi et al. 2022). We have shown that AMD RPE EV contents mirror the parental cells’ dysmetabolism at the genetic, proteomic and lipidomic levels, and that EV‐mediated signalling transfers disease features to unaffected cells in culture. This suggests that the ocular tissue has an inherent vulnerability (up to 71% of which could be attributed to genetic determinants (Seddon et al. 2005; Scholl et al. 2007; Tzoumas et al. 2022; Fritsche et al. 2016; Gorman et al. 2024)), that increases with age, the strongest risk factor for AMD (Curcio et al. 2024), and is perpetuated within the retinal niche via EVs. This vulnerability may be aggravated by choroidal delivery of systemic particles carrying stress signals. Supporting this hypothesis, human primary and iPSC‐derived RPE cells develop apolipoprotein E (APOE)‐positive drusen‐like deposits when grown on porous supports (Hallam et al. 2017; Johnson et al. 2011). Strikingly, exposure of cultured RPE to human serum induces complement activation and deposition of terminal complement complexes alongside other selected plasma‐derived proteins (e.g., serum amyloid P) onto the sub‐RPE deposits. This selective deposition of serum proteins on sub‐RPE drusen indicates specific protein‐protein interactions between RPE‐derived and plasma‐derived proteins (Johnson et al. 2011) or protein binding to hydroxyapatite spherules (Thompson et al. 2015).

The origin of drusen remains debated; however, current evidence points to a plausible scenario in which both RPE and choroidally delivered systemic material contribute to the accumulation of sub‐RPE debris (Bergen et al. 2019). Proteins identified in human donor drusen are not specific to RPE or photoreceptors and can also be found in blood. Notably, the presence of plasma proteins associated with inflammatory responses, including the acute phase proteins (e.g., serum amyloid P, serum amyloid A1, serum albumin, haptoglobin 1 and plasminogen) (Wang et al. 2010; Crabb et al. 2002), aligns with the findings of Johnson et al. (Johnson et al. 2011), and supports the idea that systemic circulation may contribute to drusen formation (Bergen et al. 2019). Correspondingly, results presented in this paper show significant alterations in AMD plasma extracellular particle‐derived protein abundance related to inflammation and acute‐phase reactants, including haptoglobin, complement proteins, ceruloplasmin, albumin, serotransferrin, serpin family proteins, hemopexin, alpha‐1‐acid glycoprotein, retinol‐binding protein 4, and inter‐alpha‐trypsin inhibitor heavy chains, among others. Since chronic low‐grade inflammation, characteristic of myeloproliferative neoplasms, has been found to correlate with an increased prevalence of drusen and late‐stage AMD in these patients compared to the general population, it may be speculated that drusen formation is influenced or even initiated by systemic inflammation (Liisborg et al. 2022, 2020). Given that Bruch's membrane shares structural similarities with the vessel wall and that inflammation is central to vascular disease, a mechanistic commonality between drusen and atherosclerotic plaque formation has been proposed (Curcio et al. 2009, 2011; Bergen et al. 2019; Kong et al. 2022). Thus, systemic inflammation and the conceptual model of “meet, greet and stick” for sub‐RPE deposits formation could potentially explain the major contribution of plasma proteins to drusen, and highlight the clinical relevance of systemic blood components in drusen formation (Bergen et al. 2019; Liisborg et al. 2022). EVs/extracellular particle aggregation could hypothetically play an important role in this process, for example, through the formation of pathologic calcifying material, as suggested by emerging evidence (Hutcheson et al. 2016; New et al. 2013; Blaser et al. 2023).

Besides proteins, lipids constitute the second major component of drusen, accounting for at least 40% of druse content (Wang et al. 2010). It has been proposed that this high lipid concentration could be attributed to lipoprotein particle retention, that is secreted by the RPE, as well as those of hepatic or intestinal origin. This is supported by the detection of immunoreactivity for certain apolipoproteins (APOA‐II and C‐III) in drusen, in the absence of mRNA synthesis by the RPE (Wang et al. 2010; Curcio et al. 2011; Li et al. 2006). Proteomic and histological studies have demonstrated a common occurrence of various apolipoproteins (E, J, C‐1, B, A‐1, A‐2, A4, C‐2 and C3) in human drusen specimens (Crabb et al. 2002; Wang et al. 2010; Li et al. 2006). Consistently, APOE, a cholesterol carrier, is also a prominent constituent of sub‐RPE deposits generated by RPE cells cultured on porous supports (Johnson et al. 2011; Hallam et al. 2017). The ultrastructure of these drusen‐like deposits resembles that observed in human donor specimens with both electron‐dense membranous material and particles/vesicles 60–80 nm in diameter, consistent with the morphology of lipoprotein particles, visible by TEM (Johnson et al. 2011; Curcio et al. 2011; Burns and Feeney‐Burns 1980). Based on Ingenuity molecular networks analysis of known drusen components, it has been proposed that APOA1 and APOA4 “are most likely derived from the blood, and not from the retina” (Bergen et al. 2019). Our proteomics analysis did not detect statistically significant changes in apolipoproteins abundance in AMD EV‐enriched preparations; however, APOA4 showed a log2 fold change of −0.6, though this did not reach statistical significance. Importantly, our sample preparation protocol, which employed size exclusion chromatography, was designed to deplete lipoprotein carry over. Therefore, our results may not be representative of quantitative comparisons of lipoprotein abundance in AMD and control plasma.

The circulating chemical fingerprint of oxidative stress damage in proteins and lipids in AMD plasma, determined by Raman spectroscopy in this study, is reminiscent of AMD drusen, Bruch's membrane and choroid signatures consisting of oxidatively modified proteins and lipids (Crabb 2014; Crabb et al. 2002). Our findings align closely with the hypothesis that “oxidative protein modifications are causally involved in drusen formation” (Crabb et al. 2002). We have previously provided strong evidence that oxidative stress mechanisms operate in cultured iPSC‐RPE with inherent vulnerabilities, such as those carrying *CFH* risk polymorphisms (Hallam et al. 2017; Kurzawa‐Akanbi et al. 2022). The second major genetic predisposition factor in *ARMS2/HTRA1* is also associated with increased vulnerability of cultured RPE to oxidative stress and a protective effect of antioxidants sodium phenylbutyrate and nicotinamide (Chang et al. 2023; Saini et al. 2017). Importantly, we demonstrated that the “oxidative stress” molecular signature of AMD iPSC‐RPE is encapsulated in EVs released by these cells and is biologically functional, capable of inducing oxidative stress responses and cellular damage in recipient retinal cells (Kurzawa‐Akanbi et al. 2022). In the current study, we show that peripheral blood plasma‐derived samples containing EVs and co‐isolated particles of the same size (overall < ∼600 nm in diameter based on TRPS and TEM, with the majority ∼120 nm, presumably lipoproteins and platelet derived vesicles), are clearly distinguishable from those of age‐matched controls and display features of oxidatively damaged proteins and lipids, and depletion of protective factors (e.g., GPX3). Taken together, these findings suggest that drusen accumulation and AMD development may result from interconnected processes—inherent retinal susceptibility and systemic dysfunction.

As the liver is the source of numerous proteins found in plasma EVs, particularly the complement proteins, albumin and most coagulation cascade proteins (Muraoka et al. 2022), the compositional abnormalities observed in AMD plasma extracellular particles, might be suggestive of liver dyshomeostasis. Additionally, the sphingolipid imbalance we identified in circulating EV‐enriched samples may also support the possibility of liver injury. Dysregulated sphingolipid metabolism, characterised by significantly increased sphingomyelin (SM 34:1) and decreased ceramides, has been linked to liver fibrosis in human patient samples and mouse models of fibrosis (Gruevska et al. 2025). Interestingly, the changes in sphingomyelin identified in the mouse fibrotic tissue included SM 34:1 and SM 42:2, the same species that were most significantly altered in our study. Gruevska et al. demonstrated that these lipidome abnormalities were accompanied by changes in gene expression of lipid‐related metabolic enzymes, and decreased mitochondrial function, as determined by pathway enrichment analysis. Likewise, abnormal sphingolipid levels have been found in plasma samples from patients with neovascular AMD (Zhao et al. 2023) and a Mendelian randomisation study proposed increasing serum sphingomyelins levels as the top causal trait in AMD (Julian et al. 2023). Consistent with the bioactive roles of sphingolipids in cellular survival and death, a growing body of evidence supports the role of sphingolipids in retinal health and disease, with ceramide‐mediated inflammation and cell death proposed as contributing factors in AMD (Simon et al. 2021). Therefore, the metabolic changes observed in AMD blood may reflect processes relevant to retinal disease and suggest the presence of systemic metabolic dysregulation in AMD.

Given the central role of the liver in systemic metabolism, liver dysfunction is a significant risk factor for secondary conditions, such as vascular disease, neuroretinal thinning and retinal neurodegeneration (Wu et al. 2023; Lampignano et al. 2022). Our findings indicate at least three points of convergence that support a mechanistic liver‐eye connection in the context of AMD: (1) complement regulation may be implicated as CFH, the strongest risk factor for AMD, is synthesised in both the liver and the RPE (Strunz et al. 2018); (2) lipid dysmetabolism and lipoprotein contribution to drusen development links retinal pathology to hepatic roles in lipid metabolism, and lipoprotein and apolipoprotein synthesis (Strunz et al. 2018, Li et al. 2006); and (3) abnormalities in hemopexin abundance detected in cultured AMD iPSC‐RPE and AMD plasma extracellular particles links redox processes and oxidative damage response in the RPE and the liver, which together represent the predominant (“systemic” and “local”) sources of hemopexin in the body (Tolosano and Altruda 2002). Altogether, we propose that despite heterogeneity of AMD, a holistic view on the condition suggests the involvement of systemic processes. In particular, dysregulation of complement system and lipid metabolism, pro‐inflammatory processes and oxidative stress appear to lie at the crossroad of retinal pathology and accompanying systemic disorder.

The implications of our study are congruent with earlier reports suggesting that AMD may be a manifestation of “underlying metabolic/vascular disease, with secondary neurodegeneration of photoreceptors” (Curcio 2011). In addition to the evidence presented above, AMD‐like features induced in animal models through a high fat “Western diet” either as the primary factor (Keeling et al. 2022) or in combination with genetic risks (Ding et al. 2011; Ramkumar et al. 2010), along with findings from human population studies, further strengthen this concept. Diets enriched in linoleic acid, monounsaturated fat, oleic acid, or saturated fat from higher intake of red meat, processed meat, high‐fat dairy products, French fries, refined grains, and eggs have been associated with an increased risk of AMD progression. Conversely, a decreased risk of AMD development and progression was linked to Mediterranean and Oriental diet patterns characterised by high intake of vegetables, whole grains and legumes, use of olive oil instead of butter, moderate consumption of fish, poultry and diary and a limited intake of red or processed meat (Pameijer et al. 2022; Chiu et al. 2014; Keenan et al. 2020). Intriguingly, a strong interaction has been observed between Mediterranean diet (specifically fish intake) and the protective *CFH* genotype rs10922109 (Keenan et al. 2020), indicating a significantly reduced risk of developing AMD. Sticking to a Mediterranean diet was also protective against the development of large drusen (Keenan et al. 2020). The Mediterranean diet, rich in antioxidants and omega‐3 fatty acids with known cyto‐protective, anti‐inflammatory and neuro‐protective properties, likely reflects key biological pathways contributing to AMD development. The compelling evidence for strong interaction between fish intake and a protective *CFH* genotype highlights an interplay between the main genetic determinants for AMD and nutrition, further underscoring mechanisms involving the complement system, immune and inflammatory responses (Keenan et al. 2020; Pameijer et al. 2022).

Altogether, our work implicates the systemic metabolic imbalance as a contributory factor to AMD pathogenesis. We provide novel insights into systemic extracellular particle signatures that align with established AMD mechanisms, and feature protein and lipid metabolism dysregulation, oxidative modifications and complement and immune system‐related compositional abnormalities. These pathology‐related signatures in AMD plasma‐derived EV‐enriched samples may have potential as disease biomarkers, supporting the development of minimally invasive liquid biopsy approaches for early disease detection, risk stratification, and monitoring treatment responses in at‐risk populations. Additionally, these findings, in conjunction with existing literature, suggest that dietary modulation may represent a potentially accessible adjunct strategy in AMD management, warranting investigation for its role in risk reduction.

## Author Contributions

**Marzena Kurzawa‐Akanbi**: conceptualization, data curation, formal analysis, funding acquisition, investigation, methodology, visualization, writing – review and editing, writing – original draft, validation. **Jennifer Haggarty**: data curation, formal analysis, methodology, writing – review and editing. **Carina Hansohn**: data curation, formal analysis, methodology, visualization, writing – review and editing. **Mohamed T Patel**: data curation, formal analysis, methodology, visualization, writing – review and editing. **Angela J Cree**: project administration, writing – review and editing, resources. **Peisi Teo**: data curation, formal analysis, writing – review and editing. **Milly Armstrong**: data curation, formal analysis, writing – review and editing. **Nathan Coles**: investigation, methodology, supervision, writing – review and editing. **Mark Platt**: data curation, methodology, writing – review and editing. **Helen Griffiths**: project administration, resources, writing – review and editing. **Heather J Cordell**: formal analysis, writing – review and editing. **Ahmad Khundakar**: writing – review and editing, investigation, supervision. **Pola Goldberg Oppenheimer**: investigation, methodology, supervision, funding acquisition, writing – review and editing. **Phillip Whitfield**: investigation, methodology, formal analysis, funding acquisition, writing – review and editing. **Andrew Lotery**: investigation, funding acquisition, writing – review and editing. **Henning Urlaub**: investigation, methodology, formal analysis, supervision, writing – review and editing. **Sina Mozaffari‐Jovin**: investigation, methodology, data curation, formal analysis, supervision, visualization, writing ‐ original draft, writing – review and editing. **Majlinda Lako**: conceptualization, formal analysis, funding acquisition, investigation, methodology, project administration, resources, visualization, writing – original draft, writing – review and editing.

## Funding

Macular Society, UKRI Frontier Research Guarantee (EP/Y031016/1) and the NIHR Moorfields Biomedical Research Centre.

## Ethics Statement

The study was approved by the London—Riverside Research Ethics Committee with a REC reference 20/PR/0772. Plasma samples were collected previously as part of ethically approved studies and with consent for use in other ethically approved studies on AMD (REC Ref 374/02/t, REC Ref 150/03/t, REC Ref 09/H0504/67).

## Conflicts of Interest

The authors report no conflict of interest.

## Supporting information




**SUPPLEMENTARY FIGURE 1: EV marker analysis in EV versus EV‐depleted fractions from size exclusion chromatography of plasma**. A size exclusion chromatography column (qEV original gen2 70 nm) was used, and the following fractions were collected post 2.9 mL default buffer volume: (EVs) 1.6 mL (4×0.4 mL)—purified collection volume, (1) to (5)—referring to 5 subsequent collections of 1.6 mL volume each. All collected volumes were concentrated to approximately 0.4 mL using Amicon ultrafiltration units 10 kDa MWCO with regenerated cellulose (Millipore). Seven or 8 µg of total protein content were analysed for marker expression by Western blotting. Notable enrichment of EV‐specific markers CD9 and CD63 was characteristic for the EV fraction, indicating high efficiency of the isolation procedure. Co‐isolation of apolipoproteins and CD41‐positive vesicles, common confounders of blood EV analysis, was however, apparent. **SUPPLEMENTARY FIGURE 2: Global plasma EV‐enriched samples proteomics in AMD versus controls**. Non‐supervised hierarchical clustering of all significantly changed proteins (cutoff 1.5‐fold change). Grey indicates undetected proteins in a sample. (A, B) Proteins upregulated and downregulated in AMD compared to controls, respectively. **SUPPLEMENTARY FIGURE 3. STRING analysis of protein‐protein interactions**. (A) A strong protein‐protein interaction (PPI) network was characteristic for the downregulated proteins whereas no network among upregulated proteins was detected. (B) PPI network analysis showing association between significantly regulated proteins of the blood coagulation and complement pathways, proteins with endopeptidase inhibitor activity and AMD‐associated proteins. PPI enrichment value of the networks, p<1.0 e‐16. **SUPPLEMENTARY FIGURE 4. Evaluating the effect of plasma thawing on EV‐enriched sample protein contents**. Total number of proteins detected in proteomics analysis of EV‐enriched samples derived from slow‐ versus rapid‐thawed plasma. (A) Statistical analysis was performed by means of Shapiro‐Wilk test for normality, followed by unpaired t test with Welch's correction in GraphPad Prism v11.0.2 (92). (B) Statistical analysis was performed by Shapiro‐Wilk test for normality, followed by Ordinary one‐way ANOVA with Tukey's multiple comparisons test in GraphPad Prism v11.0.2 (92). Ns‐not statistically significant. Mean with SEM shown. **SUPPLEMENTARY FIGURE 5. Lipidomics data analysis adjusted for the effects of *CFH* rs1061170 genotype, *HTRA1/ARMS2* rs10490924 genotype and smoking (ever smoked)**. Adjusted abundances of the identified significant features, in both positive (A) and negative ion mode (B), adjusted for the effects of *CFH* rs1061170 genotype, *HTRA1/ARMS2* rs10490924 genotype and smoking (ever smoked) were generated. The adjusted lipid measurements were calculated as the residuals from a multiple linear regression analysis of the original lipid measurements on the covariates, with genotype considered as a 3‐level factor for both *CFH* and *HTRA1/ARMS2*. The adjusted measurements were taken forward to a two‐way ANOVA with Šídák's multiple comparisons test in GraphPad Prism v11.0.2 (92) software in the same way as the original measurements. **** Indicates p < 0.0001. Data for one individual was not included in the analysis due to not having a record of whether they ever smoked. Mean with SEM shown.

**Supplementary Table 1**: Detailed clinical information on the study cohort. LE‐ left eye, RE—right eye. **Supplementary Table 2**: Primary and secondary antibodies used in Western blot analysis. **Supplementary Table 3**: Proteomics of extracellular vesicle‐enriched samples. **Supplementary Table 4**: Significantly changed proteins with 1.5‐Fold Change in abundance. **Supplementary Table 5**: Pathway enrichment analysis of differentially expressed proteins in AMD vs Controls (Gene Ontology Cellular Component (GOCC) upregulated). **Supplementary Table 6**: Pathway enrichment analysis of differentially expressed proteins in AMD vs Controls (Gene Ontology Cellular Component (GOCC) downregulated). **Supplementary Table 7**: Pathway enrichment analysis of differentially expressed proteins in AMD vs Controls (Gene Ontology Biological Process (GOBP) upregulated). **Supplementary Table 8**: Pathway enrichment analysis of differentially expressed proteins in AMD vs Controls (Gene Ontology Biological Process (GOBP) downregulated). **Supplementary Table 9**: Pathway enrichment analysis of differentially expressed proteins in AMD vs Controls (Gene Ontology Molecular Function (GOMF) downregulated). **Supplementary Table 10**: Significantly changed compounds in AMD vs Control plasma EV‐enriched samples identified through global lipidomics (Positive Ion Data). **Supplementary Table 11**: Significantly changed compounds in AMD vs Control plasma EV‐enriched samples identified through global lipidomics (Negative Ion Data).

**Supporting information**: jex270178‐sup‐0003‐SuppMat.docx

## Data Availability

The data that supports the findings of this study are available in the supplementary material of this article. The mass spectrometry proteomics data have been deposited to the ProteomeXchange under the accession number PXD076119.
