# Supplementary material for: Systematic Analysis of Plasma‐Derived Extracellular Vesicle‐Enriched Samples in Age‐Related Macular Degeneration Reveals Oxidative Stress Linked to Altered Lipid and Protein Profiles
Source: J Extracell Biol. 2026 Aug 29;5(9):e70178. doi: 10.1002/jex2.70178 (PMC13525867; doi:10.1002/jex2.70178)
Supplement: Supplementary file 1 [file JEX2-5-e70178-s001.docx]

**SUPPLEMENTARY FIGURES**

**Systematic analysis of plasma-derived extracellular vesicle-enriched samples in age-related macular degeneration reveals oxidative stress linked to altered lipid and protein profiles.**

Marzena Kurzawa-Akanbi^1,2#^, Jennifer Haggarty^3^, Carina Hansohn^4^, Mohamed T Patel^5^, Angela J Cree^6^, Peisi Teo^7^, Milly Armstrong^7^, Nathan Coles^7^, Mark Platt^8^, Helen Griffiths^6^, Heather J Cordell^9^, Ahmad Khundakar^7^, Pola Goldberg Oppenheimer^5^, Phillip Whitfield^3^, Andrew Lotery^6^, Henning Urlaub^4,10^, Sina Mozaffari Jovin^4^ and Majlinda Lako^1#^.

1. Biosciences Institute, Faculty of Medical Sciences, Newcastle University, Newcastle upon Tyne, UK.
2. NIHR Newcastle Biomedical Research Centre (BRC), Newcastle upon Tyne, UK.
3. MVLS Shared Research Facilities, College of Medical, Veterinary and Life Sciences, University of Glasgow, Glasgow, UK.
4. Max-Planck-Institute for Multidisciplinary Sciences, Göttingen, Germany.
5. School of Chemical Engineering, College of Engineering and Physical Sciences, University of Birmingham, UK.
6. Clinical and Experimental Sciences, Faculty of Medicine, University of Southampton, UK.
7. School of Health and Life Science, Teesside University, UK.
8. Loughborough University, Loughborough, UK.
9. Population Health Sciences Institute, Newcastle University, Newcastle upon Tyne, UK
10. Institute of Clinical Chemistry, University Medical Center Göttingen, Göttingen, Germany.

**SUPPLEMENTARY FIGURE 1: EV marker analysis in EV versus EV-depleted fractions from size exclusion chromatography of plasma.** A size exclusion chromatography column (qEV original gen2 70nm) was used, and the following fractions were collected post 2.9 ml default buffer volume: (EVs) 1.6 ml (4x0.4 ml) – purified collection volume, (1) to (5) – referring to 5 subsequent collections of 1.6 ml volume each. All collected volumes were concentrated to approximately 0.4 ml using Amicon ultrafiltration units 10 kDa MWCO with regenerated cellulose (Millipore). Seven or 8 µg of total protein content were analysed for marker expression by Western blotting. Notable enrichment of EV-specific markers CD9 and CD63 was characteristic for the EV fraction, indicating high efficiency of the isolation procedure. Co-isolation of apolipoproteins and CD41-positive vesicles, common confounders of blood EV analysis, was however, apparent.

**SUPPLEMENTARY FIGURE 2: Global plasma EV-enriched samples proteomics in AMD versus controls.** Non-supervised hierarchical clustering of all significantly changed proteins (cut-off 1.5-fold change). Grey indicates undetected proteins in a sample. (A, B) Proteins upregulated and downregulated in AMD compared to controls, respectively.

**SUPPLEMENTARY FIGURE 3. STRING analysis of protein-protein interactions.** (A) A strong protein-protein interaction (PPI) network was characteristic for the downregulated proteins whereas no network among upregulated proteins was detected. (B) PPI network analysis showing association between significantly regulated proteins of the blood coagulation and complement pathways, proteins with endopeptidase inhibitor activity and AMD-associated proteins. PPI enrichment value of the networks, p<1.0 e-16.

**SUPPLEMENTARY FIGURE 4. Evaluating the effect of plasma thawing on EV-enriched sample protein contents.** Total number of proteins detected in proteomics analysis of EV-enriched samples derived from slow- versus rapid-thawed plasma. (A) Statistical analysis was performed by means of Shapiro-Wilk test for normality, followed by unpaired t test with Welch’s correction in GraphPad Prism v11.0.2 (92). (B) Statistical analysis was performed by Shapiro-Wilk test for normality, followed by ordinary one-way ANOVA with Tukey’s multiple comparisons test in GraphPad Prism v11.0.2 (92). Ns – not statistically significant. Mean with SEM shown.

**SUPPLEMENTARY FIGURE 5. Lipidomics data analysis adjusted for the effects of *CFH* rs1061170 genotype, *HTRA1/ARMS2* rs10490924 genotype and smoking (ever smoked).** Adjusted abundances of the identified significant features, in both positive (A) and negative ion mode (B), adjusted for the effects of *CFH* rs1061170 genotype, *HTRA1/ARMS2* rs10490924 genotype and smoking (ever smoked) were generated. The adjusted lipid measurements were calculated as the residuals from a multiple linear regression analysis of the original lipid measurements on the covariates, with genotype considered as a 3-level factor for both *CFH* and *HTRA1/ARMS2*. The adjusted measurements were taken forward to a two-way ANOVA with Šídák’s multiple comparisons test in GraphPad Prism v11.0.2 (92) software in the same way as the original measurements. **** Indicates p<0.0001. Data for one individual was not included in the analysis due to not having a record of whether they ever smoked. Mean with SEM shown.
