# Supplementary material for: Systematic Analysis of Plasma‐Derived Extracellular Vesicle‐Enriched Samples in Age‐Related Macular Degeneration Reveals Oxidative Stress Linked to Altered Lipid and Protein Profiles
Source: J Extracell Biol. 2026 Aug 29;5(9):e70178. doi: 10.1002/jex2.70178 (PMC13525867; doi:10.1002/jex2.70178)
Supplement: Supplementary file 2 [file JEX2-5-e70178-s002.docx]

**Table of contents entry**

**Systematic analysis of plasma-derived extracellular vesicle-enriched samples in age-related macular degeneration reveals oxidative stress linked to altered lipid and protein profiles.**

Marzena Kurzawa-Akanbi^*^, Jennifer Haggarty, Carina Hansohn, Mohamed T Patel, Angela J Cree, Peisi Teo, Milly Armstrong, Nathan Coles, Mark Platt, Helen Griffiths, Heather J Cordell, Ahmad Khundakar, Pola Goldberg Oppenheimer, Phillip Whitfield, Andrew Lotery, Henning Urlaub, Sina Mozaffari Jovin and Majlinda Lako^*^.

This study presents a multimodal analysis of plasma-derived extracellular vesicle (EV)-enriched samples from a cohort of 30 patients with age-related macular degeneration (AMD) and 30 controls. Our work demonstrates that protein and lipid compositional abnormalities, accompanied by oxidative stress-related changes are associated with AMD. As we identify a dysregulation of complement and immune system proteins in AMD plasma EV-enriched samples, known contributors to retinal pathology, this work highlights the potential role of systemic dysregulation in disease mechanisms.
